# The Impact of a Mobile Money–Based Intervention on Maternal and Neonatal Health Outcomes in Madagascar: Cluster-Randomized Controlled Trial

**DOI:** 10.2196/70182

**Published:** 2025-08-15

**Authors:** Lisa Bogler, Bítia Vieira, Harizaka Emmanuel Andriamasy, Zavaniarivo Rampanjato, Sebastian Vollmer, Till Bärnighausen, Andriamampianina Ralisimalala, Julius Valentin Emmrich, Samuel Knauss

**Affiliations:** 1 Department of Economics & Centre for Modern Indian Studies University of Goettingen Göttingen Germany; 2 Global Digital Last Mile Health Research Lab Charité Center for Global Health Charité - Universitätsmedizin Berlin Berlin Germany; 3 Doctors for Madagascar Antananarivo Madagascar; 4 Heidelberg Institute of Global Health Medical Faculty and University Hospital University of Heidelberg Heidelberg Germany; 5 Ministry of Public Health of the Republic of Madagascar Antananarivo Madagascar; 6 Department of Global Health and Population Harvard T H Chan School of Public Health Boston, MA United States; 7 Africa Health Research Institute Durban South Africa; 8 Centre d'études et de recherche en économie de la santé Faculté d'économie, de gestion et de sociologie University of Antananarivo Antananarivo Madagascar; 9 Doctors for Madagascar Berlin Germany

**Keywords:** mobile health, mHealth, health financing, maternal health, health wallet, mobile money, Madagascar, cluster-randomized trial

## Abstract

**Background:**

Financial barriers to accessing obstetric care persist in many low-resource settings. With increasing use of mobile phones, mobile money services appear as a promising tool to address this concern. Maternal health care is particularly suitable for a savings program using mobile money due to the predictable timing and costs of delivery. The mobile money–based Mobile Maternal Health Wallet (MMHW) intervention aimed to ease the burden of out-of-pocket expenses related to maternal health care by providing an accessible savings tool.

**Objective:**

This study aimed to assess the impact of the MMHW on maternal and neonatal health outcomes.

**Methods:**

We used a stratified cluster-randomized trial to assess the impact of the MMHW on maternal and neonatal health outcomes in the Analamanga region of Madagascar. All 63 eligible public sector primary care health facilities (*Centres de Santé de Base* [CSBs]) within 6 strata were randomized to either receive the intervention or not. We estimated intention-to-treat effects and contamination-adjusted effects following an instrumental variable approach. The primary outcomes included (1) delivery at a health facility, (2) antenatal care visits, and (3) total health care expenditure. Between March 2022 and December 2022, a total of 6483 women who had been pregnant between July 2020 and December 2021 were surveyed.

**Results:**

Among women in catchment areas of treated CSBs, 38.79% (1297/3344) had heard of the MMHW, and 37.42% (485/1296) of them registered for the tool. There was considerable variation in uptake across treated CSBs. Descriptively, women in the catchment areas of treated CSBs were more likely to deliver in a facility and had more antenatal care visits and higher total health expenditures compared to women in control CSB catchment areas in the intention-to-treat and contamination-adjusted analyses. However, none of the effects were statistically significant.

**Conclusions:**

While this study did not identify a statistically significant impact, the estimated contamination-adjusted effects suggest that the MMHW has potential to improve access to maternal care for women who are receptive to such a mobile money–based savings tool. Estimated population-level effects were much smaller, and this study was underpowered to detect such effects due to lower-than-anticipated uptake of the intervention.

**Trial Registration:**

German Clinical Trials Register DRKS00014928; https://www.drks.de/search/de/trial/DRKS00014928

**International Registered Report Identifier (IRRID):**

RR2-10.1186/s13063-021-05694-8

## Introduction

### Background

In several sub-Saharan African countries, health care continues to be primarily financed through out-of-pocket payments, exposing households to the risk of catastrophic health expenditures and impoverishment [1,2]. Household savings can mitigate the financial strain caused by unforeseen health care costs [1,3]. However, economically disadvantaged households frequently encounter unexpected and irregular expenses that hinder their efforts to achieve long-term saving goals [4]. Consequently, obstetric care access is often hindered by limited financial resources [5]. Expectant mothers from low- and middle-income households frequently refrain from seeking birth assistance or emergency obstetric care due to the substantial expenses associated with childbirth [6,7].

In Madagascar, ensuring access to and acceptance of health care services for pregnant women remains an ongoing challenge. The national health policy of Madagascar stipulates the free provision of antenatal care (ANC) and uncomplicated deliveries in all public health care facilities. Nevertheless, charges are often imposed for folic acid, iron supplements, laboratory tests, medications, and treatments, as well as for complications during delivery. These charges act as barriers limiting women’s access to quality maternal care, particularly for those in economically disadvantaged circumstances [8,9]. The maternal mortality rate in 2020 was 392 per 100,000 live births [10] but is estimated to be up to 3 times higher in the poorest districts of the country [11]. Only approximately half of pregnant women in Madagascar complete 4 ANC visits as recommended by the World Health Organization, and over half of deliveries take place without qualified personnel [12].

Over the past 10 years, there has been astonishing growth in mobile phone subscription rates and mobile money (MM) accounts in Madagascar, as in several other sub-Saharan African countries [13-15]. MM, a commonly used term for mobile payment systems, allows individuals to securely store, send, and receive electronic money through a digital platform operated by a mobile phone carrier, serving as a viable alternative for physical cash. By leveraging simple technologies such as unstructured supplementary service data, these MM services can facilitate monetary transactions even in the absence of a traditional bank account, internet connectivity, or advanced smartphone features. It has been shown that providing a secure place for storing money can increase savings [16]. MM services could act as such a simple, accessible tool.

MM platforms do offer users the option to sign up for additional financial services, such as savings, credit, or insurance, either through mobile operators or third-party service providers. Given that nearly 80% of adults in sub-Saharan Africa lack access to formal banking services, economies in the region are increasingly reliant on mobile payment systems [17]. This technological advancement is also being harnessed to introduce mobile payment–based hospital insurance or savings mechanisms, allowing economically challenged households to allocate funds specifically for health care needs [14,18,19]. The swift accessibility of cash, remittances, electronic savings accounts, and insurance plans provided by MM is driving its growing use in the health care sector across sub-Saharan Africa [17]. MM users have a lower risk of catastrophic health care expenses during emergencies and are less prone to reducing expenditures on education or food [20-24]. This presents an opportunity for inclusive solutions to address health care coverage gaps.

Maternal and childbirth-related health care emerges as a particularly suitable candidate for a savings program due to its relatively predictable timing and costs.

In Madagascar, a significant proportion of pregnant women rely on personal cash savings to financially prepare for giving birth in health care institutions. However, this approach is particularly arduous for impoverished populations [8]. Addressing this issue, the nongovernmental organization Doctors for Madagascar developed a tool known as the Mobile Maternal Health Wallet (MMHW) to ease the burden of out-of-pocket expenses related to maternal health care [25]. Accessible via the unstructured supplementary service data menu on any mobile phone, this software platform offers expectant mothers the capability to save, pay, and receive MM and electronic vouchers for maternal health care services at participating health care providers. The personal information and funds stored in the MMHW are linked to an individual SIM card, which functions on any type of mobile device regardless of whether it is a smartphone or has internet connectivity. Additional incentives provided through the MMHW encompass access to free ambulance services in case of referrals and a free obstetric ultrasound checkup. To encourage pregnant women to save using the MMHW, Doctors for Madagascar offers 50% matching to all deposits made. Collaborating with the Malagasy Ministry of Public Health, Doctors for Madagascar randomly selected 31 out of 63 public primary care health facilities and 4 reference hospitals in Antananarivo, located in the Analamanga region of central Madagascar, for the implementation of the MMHW. To facilitate the integration of this digital payment system within health care facilities, tablets or mobile phones were distributed to the health care providers operating in the participating facilities.

### Objectives

In this study, we evaluated the impact of the MMHW intervention on maternal and neonatal health outcomes among women living in the catchment area of the health facilities. The evaluation strategy closely follows the analysis strategy outlined in the published study protocol [26].

## Methods

### Study Design

This cluster-randomized controlled trial was implemented in 3 districts of the Analamanga region in Madagascar (Antananarivo Renivohitra, Atsimondrano, and Avaradrano). Eligible for inclusion in the trial were all 63 public sector primary care health facilities (*Centres de Santé de Base* [CSBs]) that provided ANC at the time of the intervention. Due to budget constraints, not all of these could receive the intervention. Therefore, CSBs were randomized within 6 strata to either receive the intervention or not. The control group consisted of 32 CSBs, and the intervention group consisted of 31 CSBs as well as 4 public reference hospitals for maternal care. All health facilities in the intervention group received the intervention package by May 2020. Since then, registration for MMHW was open, and activities to encourage registration were ongoing until December 2022.

For the evaluation of the intervention, we used several data sources. A quantitative population-based survey was conducted during household visits between March 2022 and December 2022. For this survey, women who had completed their pregnancy between July 1, 2020, and December 31, 2021, were interviewed. They answered questions about socioeconomic characteristics, pregnancy, delivery, expenses, saving behavior, decision-making, postpartum depression, and their use of the MMHW. In addition, in households in which there was a woman matching the eligibility criteria but she was not present at the time of the visit, a short interview was conducted with 1 household member present to capture socioeconomic characteristics. This allowed us to compare the sample of women interviewed with those women who were absent but matched the eligibility criteria. The second data source was health facility records. Between August 2022 and September 2022 (with 3 exceptions for whom data were gathered in April 2023), a survey of all health facilities included in the randomization was conducted. Health facility staff was asked to share records on ANC visits, deliveries, and expenses from 2020 and 2021. In addition, respondents at facilities where the MMHW was implemented answered questions about their use of and satisfaction with the MMHW.

This trial was registered with the German Clinical Trials Register (identifier: DRKS00014928) on March 12, 2021. Registration took place after randomization and intervention implementation but before the start of data collection and outcome assessment. A study protocol outlining the evaluation design and estimation approach followed in this analysis was submitted on March 22, 2021, and published in October 2021 [26].

### Participants

For inclusion in the randomization, all CSBs in the study region that provided ANC at the time of the intervention were eligible. Eligibility was determined based on 2017 records obtained from health authorities. In addition, the 4 public reference hospitals for maternal care in Antananarivo, as defined by the Malagasy Ministry of Public Health, were included in the intervention group. Each health facility represented 1 cluster.

All health facilities included in the randomization, as well as the reference hospitals, were included in the survey of health care providers.

For the quantitative population survey, we randomly selected 4 census enumeration areas (*fokontany*) in each catchment area of the included health facilities. In catchment areas with ≤4 *fokontanys*, all were included for data collection. In each *fokontany*, data collectors mapped out the area and knocked on every second door to identify women eligible for survey participation. Women were eligible for survey participation if they had completed their pregnancy between July 1, 2020, and December 31, 2021; were aged ≥18 years; and provided verbal consent to the interview.

### Randomization and Masking

The unit of randomization for the intervention was the CSB. There was previous evidence suggesting that ANC and delivery quality varied significantly with a facility’s patient volume and capacity to perform deliveries [27]. Therefore, all health facilities were stratified into 6 subgroups according to the facility’s ANC visit volume (<1750, 1750-3500, and >3500 ANC visits per month in 2017) and the capacity to perform deliveries (none vs any deliveries). Within each stratum, the facilities were sorted in descending order of reported ANC visits. A senior biostatistician then performed pairwise randomization. Pairing 2 health facilities following each other in the ordered list, one was randomly assigned to the intervention and the other to the control group. In addition, 4 reference hospitals were assigned to receive the intervention to ensure that women could use the MMHW in case of referral for complications. Due to their nonrandom assignment to the intervention group, reference hospitals were excluded from the analysis of facility-level outcomes. As they do not have their own defined catchment areas as CSBs have from where women respondents were sampled, the reference hospitals do not affect the analysis of individual-level outcomes. During the intervention rollout, 3 CSBs assigned to the intervention group turned out not to have the technical capacity for the intervention. Of these 3 CSBs, 2 (67%) did not have the required internet connection, and the third (33%) did not manage any pregnancies. Therefore, 2 additional CSBs that were semiprivate and, therefore, ineligible for trial inclusion in 2017 and became fully public only in 2019 were added to the intervention group. These 2 additional CSBs were excluded from analysis as their assignment to the intervention group was not random. At the individual level, women living in the catchment area of these 2 CSBs were excluded from analysis. Due to the nature of the intervention, neither health facility staff nor patients visiting the facilities were blinded to intervention allocation.

### Procedures

The MMHW package consisted of 2 major components. The main component was directed at pregnant women attending ANC visits at health care facilities. Women who registered with the MMHW received a SIM card with which they were able to save in the MMHW and to pay using these savings at the health care facilities in the intervention group. Deposits could be made and used at any time during pregnancy and up to 30 days after childbirth. Additional benefits were provided for those who registered—costs for preventive drugs such as iron and folic acid tablets and mebendazole received during ANC visits, as well as costs for transfer via ambulance in the event of obstetric and neonatal emergencies to the reference hospitals, were covered. A savings incentive of 50% of the saved amount to be used for any pregnancy-related and newborn care up to 30 days after birth was provided through external donors. In addition, users could receive a free obstetric ultrasound checkup. Pregnant women were encouraged to register with the MMHW through a sensitization campaign. Sensitization took place in communities through awareness-raising mass sensitization events and home visits to pregnant women by community health workers and during group meetings organized by workers of Doctors for Madagascar at health care facilities during the facilities’ official dates for ANC visits.

The second component was directed at CSBs and provided maternal and neonatal health training, capacity-building, and refresher courses for health workers in maternity wards and ANC departments. The trainings were conducted by trainers from the Ministry of Public Health. The goal of these trainings was to ensure quality of care matching the expected increased demand for health care services. Financial incentives to participate in the intervention were also provided. For each delivery of a beneficiary, the health care professional in charge of the delivery received a bonus of MGA 10,000 (US $2.44 according to the OANDA currency converter rate for January 1, 2021). The pharmacists responsible for taking photo evidence of claims received a bonus of MGA 2000 (US $0.51) per patient and month for each claim sent via the MMHW platform. In addition, medical equipment, including blood pressure monitors, stethoscopes, examination beds, bedpans, and baby scales, was distributed to the corresponding maternity units.

### Outcomes

In this study, we analyzed the outcomes as described in the published study protocol, which are based on the quantitative household survey and the health facility records [26]. Primary outcomes were (1) facility-based delivery; (2) number of ANC visits per woman; and (3) total health expenditure during pregnancy, delivery, and the neonatal period per woman. Secondary outcomes at the individual level were related to ANC (number of diagnoses received and whether they had ultrasound checkups or received iron and folic acid tablets or syrup), the delivery process (whether they had complications, planned or emergency cesarean section, or neonatal mortality), finances (health savings, contributions to savings, relative health care expenditure, and indication of financial distress), and satisfaction after delivery (with the health facility, the health system, and life), as well as postpartum depression score. Secondary outcomes at the facility level included maternal and neonatal mortality, number of ANC drugs distributed, and public sector costs. Compared with the full list of proposed outcomes in the protocol, we excluded those outcomes that could not be analyzed sensibly following the planned estimation approach as they would only be relevant for women who actually registered with the MMHW (ie, MMHW use, recommendation of MMHW, and time of sign-up). Furthermore, maternal mortality was only analyzed based on data from health facility records as the outcome was not captured accurately in the quantitative household survey. A full list of outcomes and details regarding their coding can be found in Tables S1 and S2 in Multimedia Appendix 1.

### Statistical Analysis

The analysis in this study followed the estimation strategy outlined in the published study protocol [26]. The primary analysis was an intention-to-treat (ITT) estimation of all outcomes. We regressed each individual-level outcome on an indicator of living in the catchment area of either a treated or control health facility and clustered SEs at the facility level. Outcomes at the facility level were regressed on an indicator of treatment assignment. For both individual- and facility-level outcomes, we added strata dummy variables to account for stratification during randomization. Because of the randomized treatment assignment, a comparison of postintervention outcomes between the intervention and control group, on average, provided the unbiased ITT effect. This ITT effect captured the real-life policy impact of the intervention. As a secondary analysis, we conducted a contamination-adjusted analysis for outcomes at the individual level using the random assignment to the intervention versus control group as an instrumental variable (IV). This estimation of the local average treatment effect provided a measure of the effect size that would have been attained without contamination assuming that those who did not take up the intervention would behave similarly once registered for the tool as those who did take up the intervention. Contamination in this case would occur if women living in an intervention area did not register for the MMHW and women living in a control area did. This is very possible in this context in which women do not necessarily choose the facility in the catchment area they live in. Actual registration with the MMHW was instrumented with treatment assignment in the IV analysis. In addition, we instrumented having heard of the MMHW and having used the MMHW for paying with treatment assignment. For ease of interpretation and comparison between ITT and IV estimation, we used linear regressions for all outcomes. We present absolute and relative effect sizes (relative to the mean of the outcome in the control group). As a robustness check, we reran estimations excluding the 3 CSBs that were assigned to the intervention but could not use it. As a further robustness check on individual-level outcomes, we reran estimations with controls for individual characteristics that were imbalanced (employment status, access to a mobile phone, and television ownership). We assessed statistical significance at the 5% level. All statistical analyses were conducted using Stata (version 16; StataCorp).

### Power Calculations

A power calculation was presented in the study protocol [26]. Minimum detectable differences between the intervention and control groups were calculated for the 3 primary outcomes using methods for cluster-randomized controlled trials. We assumed that 50% of survey participants in the catchment areas of intervention health facilities would adopt the MMHW. Power calculations showed that the study would have 80% power to detect a 4–percentage point increase in the rate of facility-based deliveries, an increase of 0.08 average ANC visits per woman, and an increase in total health expenditure of MGA 1700 (US $0.41 according to the OANDA currency converter rate for August 16, 2022) if 2300 women were surveyed per group. However, intervention uptake differed from this assumption, as discussed in the following sections.

### Ethical Considerations

This trial was approved by the Institutional Review Board of Heidelberg University on February 3, 2020 (S-428/2019). The study was conducted in accordance with the WMA Declaration of Helsinki. Informed consent was obtained orally from all study participants before their interviews. Privacy and confidentiality of survey participants’ data and identity was maintained. Survey participants received a bar of soap as a locally appropriate in-kind compensation for their time.

## Results

### Sample Description and Balance

**Figure 1:**
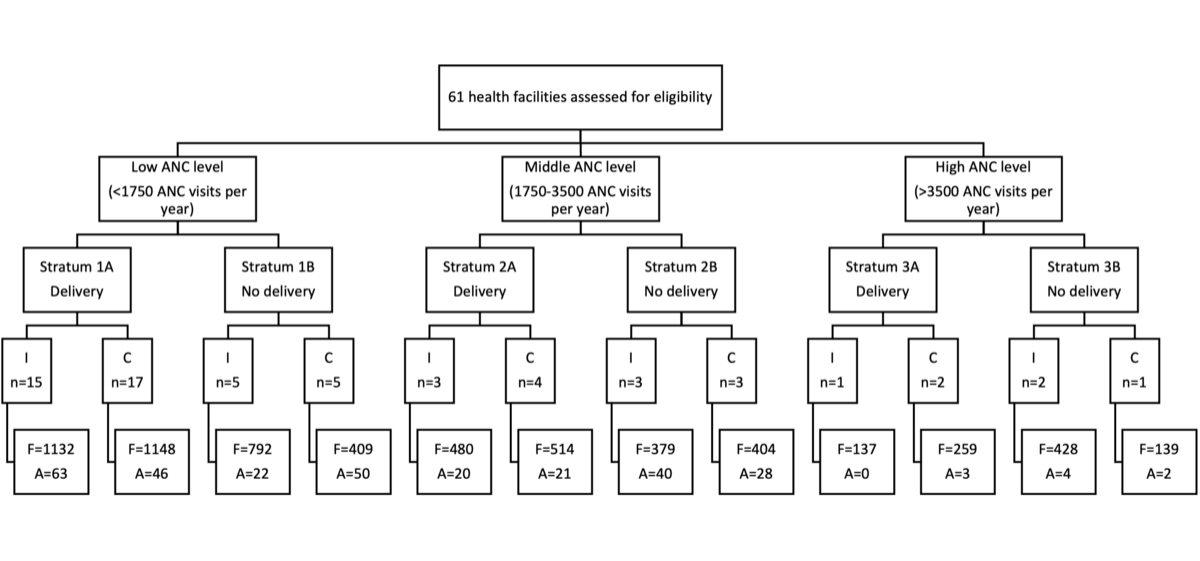
Flowchart of sample selection. A: number of interviews for absent eligible women; ANC: antenatal care; C: control group; F: number of full interviews; I: intervention group; n: number of facilities.

A total of 6323 women in the catchment areas of the CSBs in the control and intervention groups were identified as eligible for participation and present at the time of the home visit, of whom 6243 (98.7%) completed the full interview. The interview about eligible women who were not present at the time of the visit was completed by 300 respondents (ie, household members who were present and could answer questions capturing the basic characteristics of the eligible women). Figure 1 provides a flowchart of the sample.

Most women surveyed were aged <35 years (2673/6243, 43.82% between 17 and 24 years; 2741/6243, 43.91% between 25 and 34 years) with 1 or 2 children (2282/6235, 37% with 1 child; 1990/6235, 31.92% with 2 children), and the large majority was married (see Table 1 for a summary of socioeconomic characteristics). Some women reported no children as their children had passed away and the eligibility criteria were based on the timing of past pregnancies and not childbirth. A large share of women (4437/6240, 71.11%) had completed secondary or higher education, but less than half (3061/6230, 49.13%) reported receiving regular income. Almost half (2820/6242, 45.18%) were currently not working, which was likely driven by the fact that these were women with very young children. Access to a mobile phone, a requirement for using the intervention, was common but not universal as 58.09% (3626/6242) reported having access to a mobile phone and 77.96% (2827/3626) of those with access reported that the phone was their own. The large majority of respondents (5634/6239, 90.3%) were not covered by any health insurance.

**Table 1:** Sample characteristics at the individual level (N=6243)^a^.

Characteristic			Full interviews											Absent sample				
			Number of observations per characteristic, n(%)		Women in the total sample, n (%)		Women in the control group, n (%)		Women in the intervention group, n (%)		*P* value		Number of observations per characteristic, n (%)			Women, n (%)		*P* value
**Age of the woman (y)**			6243 (100)										299 (99.7)					
	17-24			2673 (42.82)		1260 (43.52)		1413 (42.2)		.29					97 (32.4)		<.001	
	25-34			2741 (43.91)		1281 (44.25)		1460 (43.61)		.61					157 (52.5)		.003	
	35-55			829 (13.28)		354 (12.23)		475 (14.19)		.02					45 (15.1)		.38	
Married			6242 (99.98)		5680 (91)		2649 (91.5)		3031 (90.56)		.19		—^b^			—		—
**Number of children**			6235 (99.87)										—			—		—
	0			17 (0.27)		7 (0.24)		10 (0.3)		.67								
	1			2282 (36.6)		1092 (37.77)		1190 (35.59)		.07								
	2			1990 (31.92)		915 (31.65)		1075 (32.15)		.67								
	3			1098 (17.61)		489 (16.91)		609 (18.21)		.18								
	4			495 (7.94)		229 (7.92)		266 (7.95)		.96								
	≥5			353 (5.66)		159 (5.5)		194 (5.8)		.61								
**Level of education**			6240 (99.95)										—			—		—
	No education			104 (1.67)		47 (1.62)		57 (1.7)		.80								
	Primary education			1699 (27.23)		800 (27.63)		899 (26.88)		.50								
	Secondary education			3711 (59.47)		1749 (60.41)		1962 (58.65)		.16								
	Higher education			726 (11.63)		299 (10.33)		427 (12.77)		.003								
Received regular income			6230 (99.79)		3061 (49.13)		1398 (48.32)		1663 (49.84)		.23							
**Type of employment**			6242 (99.98)										—			—		—
	Wage earner			357 (5.72)		157 (5.42)		200 (5.98)		.35								
	Self-employed			2152 (34.48)		915 (31.61)		1237 (36.96)		<.001								
	Family farm, livestock, or fishing			899 (14.40)		572 (19.76)		327 (9.77)		<.001								
	Student			29 (0.46)		14 (0.48)		15 (0.45)		.84								
	Not working			2820 (45.18)		1257 (43.42)		1563 (46.7)		.009								
	Other			131 (2.10)		65 (2.25)		66 (1.97)		.45								
Access to a mobile phone			6242 (99.98)		3626 (58.09)		1632 (56.39)		1994 (59.56)		.01		300 (100)			204 (68)		.001
**Phone ownership**			3626 (58.08)										—			—		—
	Own phone			2827 (77.96)		1255 (76.90)		1572 (78.84)		.14								
	Spouse’s phone			642 (17.71)		298 (18.26)		344 (17.25)		.38								
	Someone else’s phone			157 (4.33)		79 (4.84)		78 (3.91)		.17								
Used mobile money (excluding mTOMADY)			5152 (82.52)		2371 (46.02)		1218 (46.68)		1153 (45.34)		.33							
Television ownership			6231 (99.81)		3184 (51.10)		1306 (45.24)		1878 (56.16)		<.001		300 (100)			204 (68)		<.001
**Vehicle ownership**			6234 (99.86)										298 (99.3)					
	No vehicle			4808 (77.13)		2201 (76.16)		2607 (77.96)		.09					214 (71.8)		.03	
	Bicycle			824 (13.22)		418 (14.46)		406 (12.14)		.007					32 (10.7)		.22	
	Motorcycle			568 (9.11)		268 (9.27)		300 (8.97)		.68					39 (13.1)		.02	
	Car			175 (2.81)		84 (2.91)		91 (2.72)		.66					29 (9.7)		<.001	
	Other vehicles			46 (0.74)		24 (0.83)		22 (0.66)		.43					3 (1)		.60	
**Insurance**			6239 (99.94)										—			—		—
	None			5634 (90.30)		2620 (90.53)		3014 (90.1)		.57								
	Community-based medical health insurance			36 (0.58)		13 (0.45)		23 (0.69)		.22								
	Microfinance-based scheme			4 (0.06)		1 (0.03)		3 (0.09)		.39								
	Employment-based scheme			429 (6.88)		202 (6.98)		227 (6.79)		.76								
	Association or NGO^c^			32 (0.51)		12 (0.41)		20 (0.6)		.31								
	Other			111 (1.78)		48 (1.66)		63 (1.88)		.50								

^a^This table shows the shares of respondents for each characteristic in percentages, for the sample who took part in the full interviews (total sample, control, and intervention), and the sample who gave interviews for women absent at the time of the visit. The columns Number of observations per characteristic, n (%) provide the number of women in the respective sample for whom the information was available. The *P* values for the full interviews correspond to a 2-tailed *t* test of the difference between the control and intervention group. The *P* values for absent interviews correspond to a 2-tailed *t* test of the difference between the interviews about absent women and the full interviews.

^b^Information not available for absent sample.

^c^NGO: nongovernmental organization.

The sample of respondents who completed the full interview slightly differed from the eligible women who were absent at the time of the visit (Table 1). Absent women were less likely to be aged <25 years and more likely to be aged 25 to 34 years than women present at their household residence; more likely to have access to a mobile phone; and more likely to own a television, motorcycle, or car. This suggests that absent women were more likely to be working, possibly because it had not been their first birth as they were older and they were slightly wealthier.

Randomization of health facilities led to a balanced sample of health facilities (Table 2) and of respondents in terms of most characteristics (Table 1). However, some statistically significant differences between the control and intervention groups emerged at the individual level, especially regarding type of employment and asset ownership. Respondents in the intervention group were more likely to be self-employed or not working and less likely to work on a family farm or in livestock or fishing. Access to a mobile phone was slightly higher in the intervention group, as was the probability of owning a television.

**Table 2:** Facility characteristics (2020 and 2021)^a^.

Characteristic	Number of observations		Values, mean (SD)			*P* value		2020				2021		
	2020	2021	2020	2021			Control		Intervention	*P* value	Control		Intervention	*P* value
Number of ANC^b^ visits	57	56	1416 (1194)	1379 (1230)	.38		1453 (1270)		1429 (1128)	.94	1297 (1275)		1468 (1197)	.61
Number of deliveries	56	50	236 (333)	209 (273)	.10		234 (393)		222 (255)	.89	201 (301)		217 (248)	.83
Maternal mortality	39	36	0.003 (0)	0.005 (0)	.31		0.002 (0)		0.004 (0)	.43	0.007 (0)		0.003 (0)	.38
Newborn mortality	38	37	0.000 (0)	0.000 (0)	.32		0.001 (0)		0.000 (0)	.30	0.000 (0)		0.000 (0)	—^c^
Number of iron and folic acid supplements distributed	55	52	38,420 (31,086)	37,904 (31,984)	.76		40,530 (36,894)		35,317 (23,407)	.54	39,339 (38,026)		36,411 (25,228)	.74
Number of iron and folic acid supplements distributed per ANC visit	54	51	34 (40)	32 (26)	.79		37 (55)		28 (2)	.41	35 (37)		28 (2)	.34
Number of antiparasite medications distributed	56	51	312 (273)	310 (273)	.88		323 (303)		289 (239)	.64	300 (289)		315 (259)	.85
Expenses for ANC drugs (MGA)	57	51	1401 (285)	1399 (292)	.32		1403 (203)		1423 (355)	.79	1395 (212)		1406 (357)	.89
Expenses for delivery drugs (MGA)	41	36	7329 (4973)	7329 (4792)	—		6887 (3954)		8238 (5802)	.39	6915 (4210)		7660 (5296)	.65

^a^Data from the health facility survey excluding 2 *Centres de Santé de Base* (public sector primary care health facilities) and 4 reference hospitals assigned to the intervention group nonrandomly. The number of observations and means in the total sample are split by year and by control and intervention group. *P* values correspond to 2-tailed t tests of the difference in means across years and treatment.

^b^ANC: antenatal care.

^c^Values for the two groups, which are being compared, are equal.

### Uptake of the MMHW Intervention

A total of 35 health facilities, including the 4 reference hospitals, received the intervention, whereas 32 facilities did not. Of the 35 health facilities that received the intervention, including the reference hospitals, 2 (6%) were dropped from the analysis because their assignment was not random. In the survey of health facilities, 3% (1/32) of the respondents in the control group reported that the MMHW was usable in their facility, whereas 12% (4/33) of the respondents in the intervention group reported that it was not usable. Of the latter, 75% (3/4) were those CSBs that could not implement the intervention due to technical issues. The fourth facility seemed to have stopped using the MMHW since intervention rollout.

Intervention uptake was lower among surveyed women than assumed in the power calculation. Among women in the catchment areas of treated CSBs, 38.79% (1297/3344) had heard of the MMHW (372/2891, 12.87% in the control group). Registration rates were at 14.51% (485/3344) in the intervention group and 3.46% (100/2890) in the control group, corresponding to 37.42% (485/1296) and 27% (100/371) of those who had heard of the MMHW, respectively. Among those who registered for the MMHW, 66.7% (320/480) in the intervention group and 65% (65/100) in the control group actually used it to pay for maternal care services. Uptake varied considerably across treated CSBs; the share of women who had heard of the MMHW ranged from 4% (1/24) to 78% (25/32), and the share of women who registered ranged from 0% (0/24) to 69% (22/32).

### Impact on Primary and Secondary Outcomes

**Figure 2:**
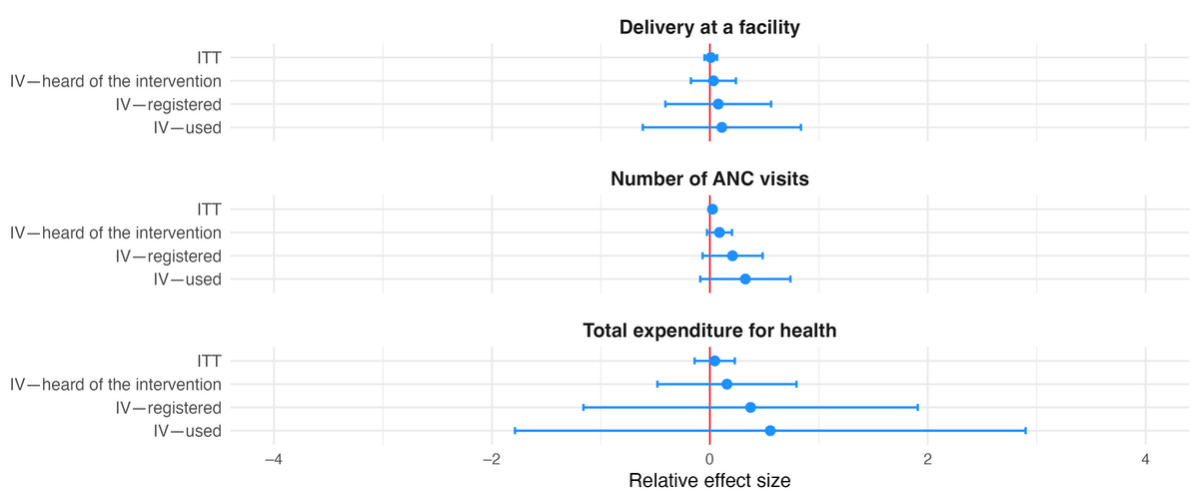
Relative effect sizes of intention-to-treat (ITT) and instrumental variable (IV) estimations for primary individual-level outcomes. The graph shows the estimated relative effect sizes with 95% CIs from the ITT analysis as well as the IV analysis where assignment to treatment was used as an instrument for having heard of the Mobile Maternal Health Wallet (MMHW), having registered with the MMHW, and having used the MMHW to pay. ANC: antenatal care.

**Figure 3:**
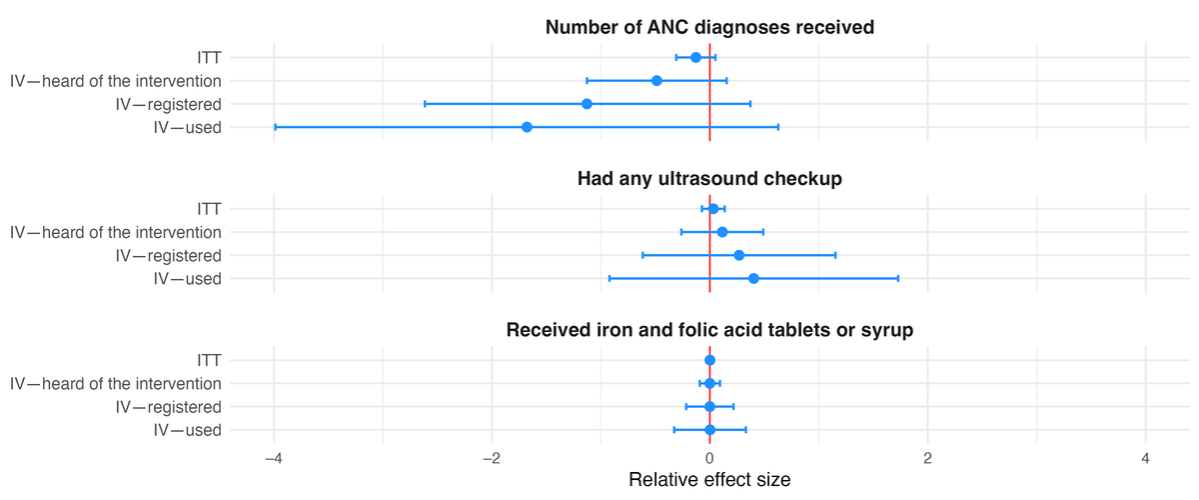
Relative effect sizes of intention-to-treat (ITT) and instrumental variable (IV) estimations for secondary individual-level antenatal care (ANC)–related outcomes. The graph shows the estimated relative effect sizes with 95% CIs from the ITT analysis as well as the IV analysis where assignment to treatment was used as an instrument for having heard of the Mobile Maternal Health Wallet (MMHW), having registered with the MMHW, and having used the MMHW to pay.

**Figure 4:**
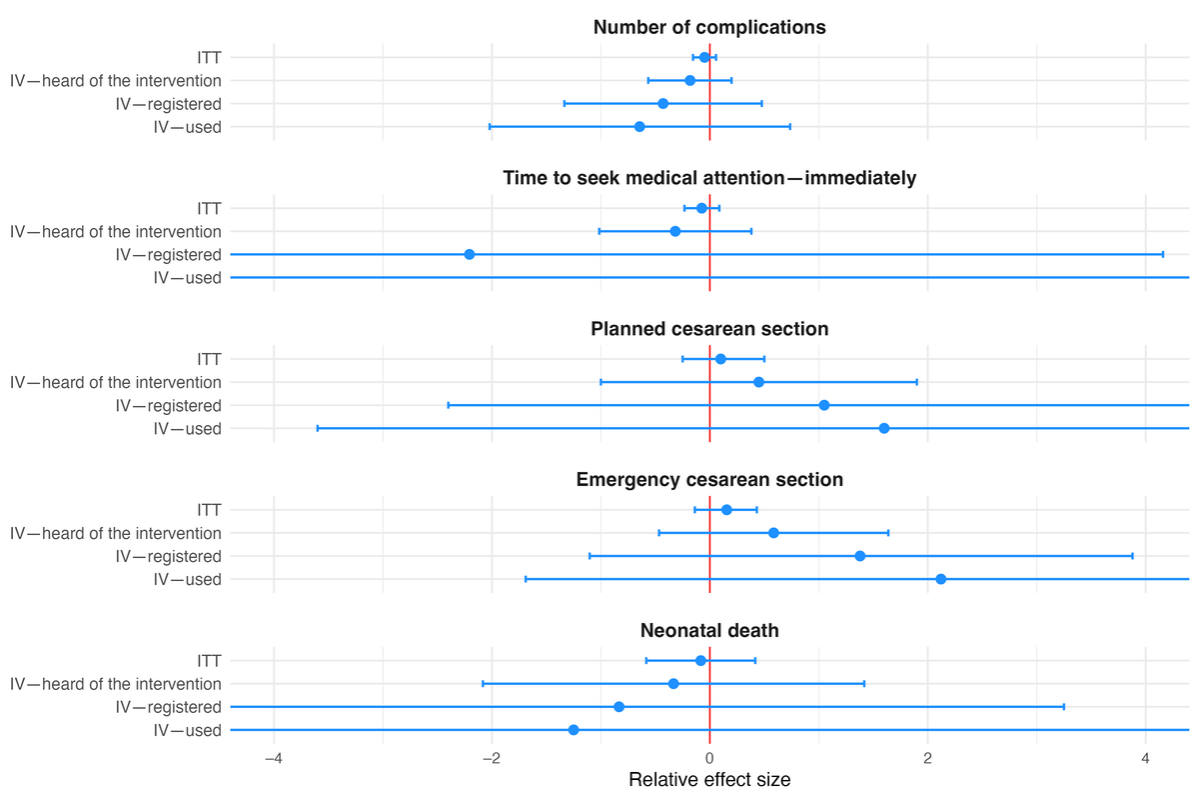
Relative effect sizes of intention-to-treat (ITT) and instrumental variable (IV) estimations for secondary individual-level delivery-related outcomes. The graph shows the estimated relative effect sizes with 95% CIs from the ITT analysis as well as the IV analysis where assignment to treatment was used as an instrument for having heard of the Mobile Maternal Health Wallet (MMHW), having registered with the MMHW, and having used the MMHW to pay. Elements not visible on the graph: time to seek medical attention in the IV—registered estimation (−2.206, 95% CI −8.573 to 4.159), time to seek medical attention in the IV—used estimation (−5.783, 95% CI −29.899 to 18.332), planned cesarean section in the IV—registered estimation (1.050, 95% CI −2.400 to 4.500), planned cesarean section in the IV—used estimation (1.600, 95% CI −3.600 to 6.850), emergency cesarean section in the IV—used estimation (2.121, 95% CI −1.690 to 5.931), neonatal death in the IV—registered estimation (−0.833, 95% CI −4.917 to 3.250), and neonatal death in the IV—used estimation (−1.250, 95% CI −7.417 to 5.000).

**Figure 5:**
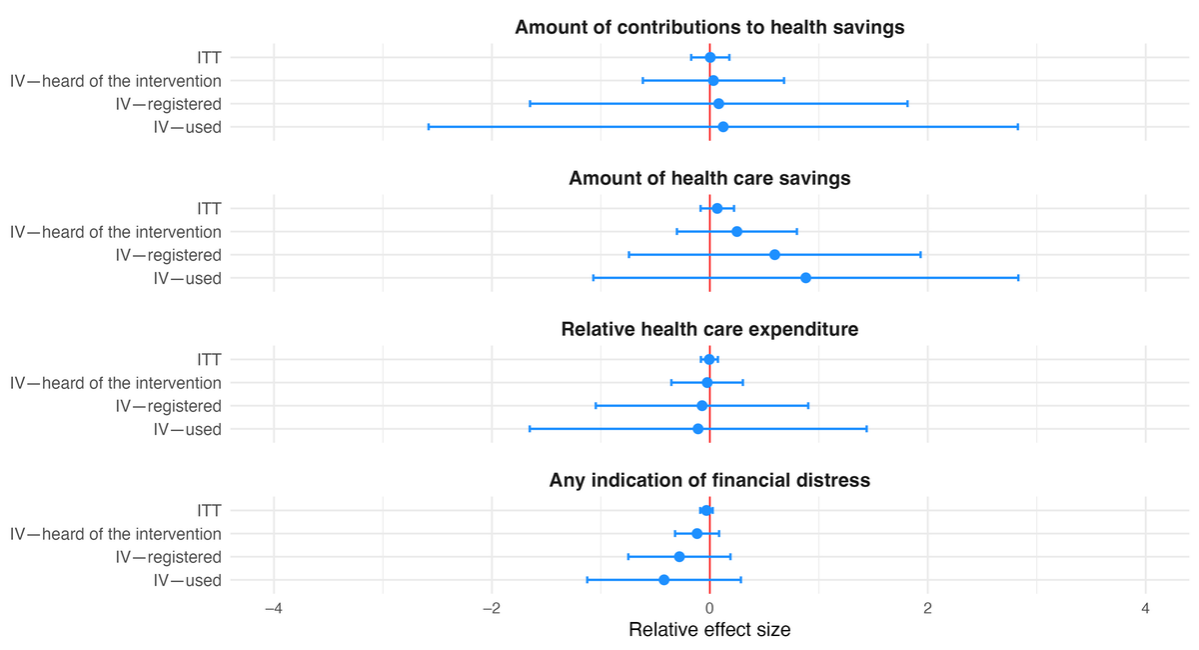
Relative effect sizes of intention-to-treat (ITT) and instrumental variable (IV) estimations for secondary individual-level finance-related outcomes. The graph shows the estimated relative effect sizes with 95% CIs from the ITT analysis as well as the IV analysis where assignment to treatment was used as an instrument for having heard of the Mobile Maternal Health Wallet (MMHW), having registered with the MMHW, and having used the MMHW to pay.

**Figure 6:**
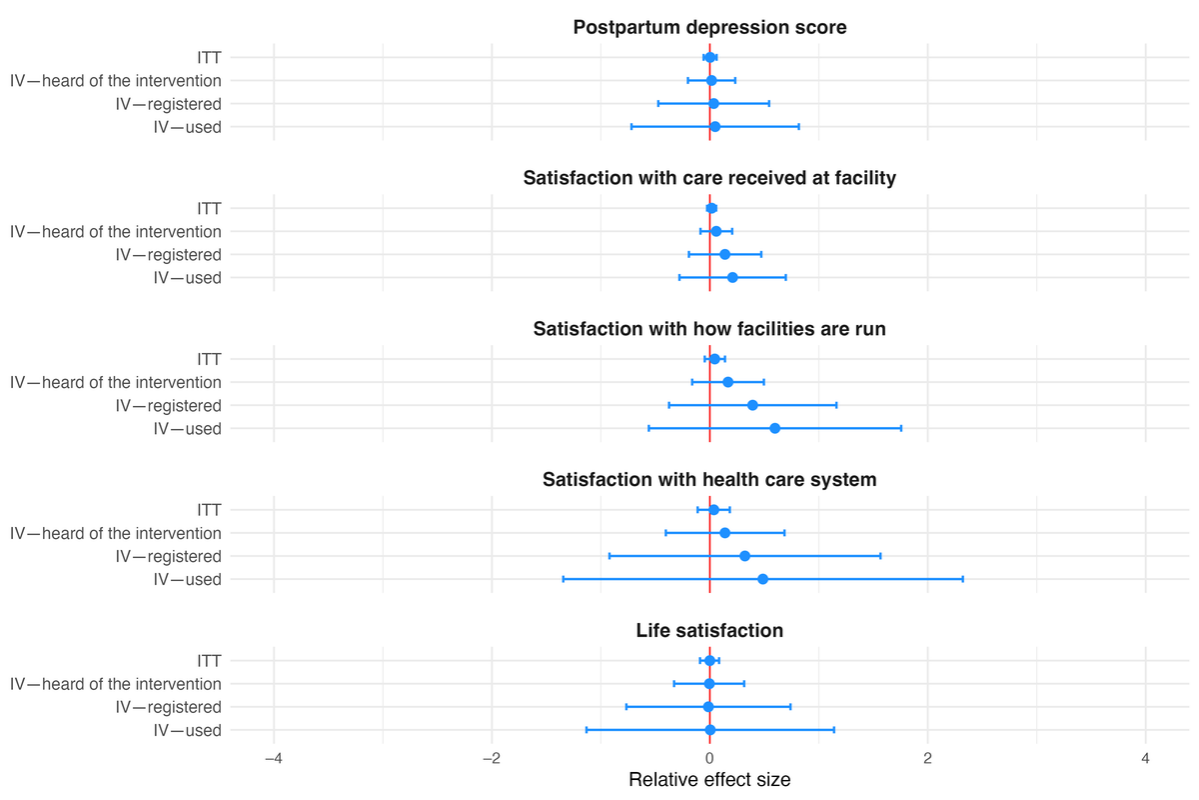
Relative effect sizes of intention-to-treat (ITT) and instrumental variable (IV) estimations for secondary individual-level satisfaction-related outcomes and postpartum depression score. The graph shows the estimated relative effect sizes with 95% CIs from the ITT analysis as well as the IV analysis where assignment to treatment was used as an instrument for having heard of the Mobile Maternal Health Wallet (MMHW), having registered with the MMHW, and having used the MMHW to pay.

Results of the ITT and IV estimations for outcomes on the individual level are shown in Table 3 for primary outcomes, Table S3 of Multimedia Appendix 1 for secondary outcomes, and are illustrated in Figures 2-6. None of the coefficients for the 3 primary outcomes—delivery at a facility, number of ANC visits, and total expenditure for health—were statistically significant. Relative effect sizes were larger in the IV analysis than in the ITT analysis, and they were larger the stricter the instrumented indicator was (Figure 2). For delivery at a facility, the relative effect size in the ITT analysis was 0.009, meaning that the share of women delivering at health facilities increased by 0.9% of the control mean (95% CI −0.049 to 0.067). When treatment was used as an instrument for having heard of the MMHW, the relative effect size increased to 0.033 (95% CI −0.174 to 0.239). When treatment was used as an instrument for having registered for the MMHW, the relative effect size was 0.077 (95% CI −0.408 to 0.562). When treatment was used as an instrument for having used the MMHW, the relative effect size was 0.110 (95% CI −0.616 to 0.836), suggesting an increase by 11% of the control mean. For the number of ANC visits, the respective relative effect sizes were 0.024 (95% CI −0.009 to 0.056) in the ITT analysis, meaning an increase of 2.4% over the control mean ANC visits, 0.088 (95% CI −0.026 to 0.203) for IV—heard of the intervention, 0.208 (95% CI −0.067 to 0.484) for the IV—registered with the intervention, and 0.326 (95% CI −0.088 to 0.739) for IV—used the intervention. For total expenditure for health, the respective relative effect sizes were 0.045 (95% CI −0.140 to 0.229) in the ITT analysis, meaning an increase of 4.5% over the control mean of total expenditure for health, 0.157 (95% CI −0.481 to 0.795) for IV—heard of the intervention, 0.374 (95% CI −1.160 to 1.908) for IV—registered with the intervention, and 0.555 (95% CI −1.789 to 2.898) for IV—used the intervention.

**Table 3:** Regression results of intention-to-treat (ITT) and instrumental variable (IV) estimations for individual-level outcomes^a^.

Outcome	Number of clusters	ICC^b^	Number of observations	Mean (SD)	ITT				IV—heard of the intervention				IV—registered for the intervention				IV—used the intervention		
					Coefficient	*P* value	Relative effect size	Coefficient		*P* value	Relative effect size	Coefficient		*P* value	Relative effect size	Coefficient		*P* value	Relative effect size
Delivery at a facility	61	0.032	6185	0.780 (0.414)	0.007	.76	0.009	0.026		.76	0.033	0.060		.76	0.077	0.086		.77	0.110
Number of ANC^c^ visits	61	0.021	6186	4.325 (1.515)	0.102	.15	0.024	0.381		.13	0.088	0.901		.14	0.208	1.408		.12	0.326
Total expenditure for health (MGA)	61	0.048	4692	208,021 (330,946)	9309	.63	0.045	32,891		.63	0.157	78,346		.63	0.374	116,198		.64	0.555

^a^This table shows the regression results of the ITT and IV estimations for primary outcomes. It reports the number of clusters and observations used for each outcome, the intraclass correlation coefficient, the mean value and SD of each outcome in the control group, the coefficient of being in the catchment area of a treated Centre de Santé de Base (CSB; public sector primary care health facility) with the corresponding *P* value, and the relative effect size (calculated as the coefficient divided by the mean) for both ITT and IV models. In all models, indicator variables for strata were included as controls, and SEs were clustered at the CSB level.

^b^ICC: intraclass correlation coefficient.

^c^ANC: antenatal care.

We grouped secondary outcomes into 4 categories. Coefficients for ANC- and delivery-related outcomes were all statistically insignificant. With the exception of the indicator for having received iron and folic acid tablets or syrup, they all followed the pattern described for primary outcomes (ie, larger relative effect sizes in the IV analysis; Figures 3 and 4). The same held true for the 4 outcomes grouped as being related to finances, including the amount of contributions to health savings, amount of health savings, relative health expenditure, and indication of financial distress (Figure 5). All coefficients for these outcomes went in the direction intended by the intervention, showing positive associations with the amount of savings and contributions and negative associations with relative health care expenditure and indication of financial distress, but no coefficient was statistically significantly different from 0 at the 5% level. The last group combined outcomes related to satisfaction and the postpartum depression score. None of the coefficients were statistically significant. Apart from the coefficient for life satisfaction, effect sizes increased across specifications, as described previously (Figure 6).

In a robustness check, we excluded those CSBs that did not implement the MMHW for technical reasons. The results are shown in Table S4 in Multimedia Appendix 1. For all primary outcomes and several secondary outcomes (whether the women had any ultrasound checkups, whether they received iron and folic acid tablets or syrup, the number of complications, the time to seek medical attention, planned and emergency cesarean sections, the amount of health care savings, and postpartum depression score), the effect sizes went in the same direction as and were larger (in absolute terms) than in the full sample, but the coefficients were still not statistically significant. For other outcomes (number of ANC diagnoses, amount of contributions to health savings, indication of financial distress, and all indicators of facility and health care satisfaction), effect sizes were smaller but of the same sign. The effect sizes for relative health care expenditure and life satisfaction went in the opposite direction to that in the main specification. The effect size for children passing away before their first birthday was still negative but closer to 0.

In a further robustness check, we controlled for variables that appeared imbalanced. The results are shown in Table S5 in Multimedia Appendix 1. For 2 of the primary outcomes, delivery at a facility and total expenditure for health, the signs of the coefficients changed, indicating a reduction from the control mean. Among several secondary outcomes (whether the women had any ultrasound checkups, planned cesarean sections, 3 of 4 finance-related outcomes, and postpartum depression score), the sign of the coefficients also changed. Again, coefficients were not statistically significant.

The results of the ITT estimations at the facility level are shown in Table 4. Regarding outcomes at the individual level, no statistically significant impact on facility-level outcomes was found for outcomes either in 2020 or 2021. Effects on the number of iron and folic acid supplements and antiparasite medications went in the opposite direction of what was intended by the intervention. In the robustness checks excluding the CSBs in the intervention group that did not implement the MMHW, there were also no statistically significant effects (Table S6 in Multimedia Appendix 1).

**Table 4:** Regression results of intention-to-treat (ITT) estimations for facility-level outcomes^a^.

Outcome			Number of observations		Values, mean (SD)		Coefficient		*P* value		Relative effect size
**Outcomes based on 2020 records**											
	Number of ANC^b^ visits	57		1429 (1128)		42		.81		0.029	
	Number of deliveries	56		222 (255)		41		.43		0.180	
	Maternal mortality	39		0.004 (0)		0.002		.42		0.667	
	Newborn mortality	38		0.000 (0)		−0.001		.34		—^c^	
	Number of iron and folic acid supplements distributed	55		35,317 (23,407)		−359		.95		−0.009	
	Number of iron and folic acid supplements distributed per ANC visit	54		28 (2)		−10		.40		−0.303	
	Number of antiparasite medications distributed	56		289 (239)		−23		.72		−0.075	
	Expenses for ANC drugs	57		1423 (355)		9		.90		0.006	
	Expenses for delivery drugs	41		8238 (5802)		2331		.08		0.308	
**Outcomes based on 2021 records**											
	Number of ANC visits	56		1468 (1197)		160		.35		0.116	
	Number of deliveries	50		217 (248)		44		.34		0.211	
	Maternal mortality	36		0.003 (0)		−0.004		.44		−0.800	
	Newborn mortality	37		0.000 (0)		0.000		—		—	
	Number of iron and folic acid supplements distributed	52		36,411 (25,228)		−3278		.50		−0.087	
	Number of iron and folic acid supplements distributed per ANC visit	51		28 (2)		−7		.37		−0.219	
	Number of antiparasite medications distributed	51		315 (259)		−14		.83		−0.046	
	Expenses for ANC drugs	51		1406 (357)		−7		.93		−0.005	
	Expenses for delivery drugs	36		7660 (5296)		952		.43		0.130	

^a^This table shows the regression results of the ITT estimations for secondary outcomes at the facility level. Centres de Santé de Base (CSBs; public sector primary care health facilities) and reference hospitals assigned to the intervention group nonrandomly are excluded. The table reports the number of observations used for each outcome, the mean value and SD of each outcome in the control group, the coefficient of being treated with the corresponding *P* value, and the relative effect size (calculated as the coefficient divided by the mean). Indicator variables for strata were included as controls.

^b^ANC: antenatal care.

^c^Calculation of *P* value or relative effect size was not possible.

## Discussion

### Principal Findings

In this analysis, we quantitatively assessed the impact of a mobile savings tool for maternal and newborn care in the Analamanga region in Madagascar. With this tool, users could save money and pay for services at participating health facilities, including primary care health facilities as well as reference hospitals. The intervention was evaluated using a cluster-randomized controlled trial.

In the ITT analysis, we did not detect any statistically significant impact of the MMHW intervention on maternal and neonatal health outcomes. The lack of statistically significant effects could be due to the study being underpowered to detect these effect sizes given that the realized knowledge about the intervention and the uptake among the survey participants was below what was assumed in the initial power calculation. It could also mean that the intervention did not achieve population-level effects.

Relative effect sizes were larger in the contamination-adjusted analysis than in the ITT analysis, and they were meaningful though also statistically insignificant, especially when treatment was used as instrument for having used the MMHW for paying. The larger effects in the contamination-adjusted analysis indicate that the intervention potentially had an impact on those who were receptive to it and used the tool. Coefficients for the 3 primary outcomes were all positive, suggesting a higher likelihood of delivery at a facility, a higher number of ANC visits, and higher health care expenditure among MMHW users. The latter could indicate that, due to financial barriers, these women previously did not purchase all the services they required, leading to an initial increase in expenditure after financial barriers were eased [9]. However, when controlling for employment status and asset ownership, the direction of the association changed for several outcomes, including delivery in a facility and health care expenditure. This could indicate a potential confounding bias in the main analysis.

A striking finding of this study was the level of expenditure for maternal care, which is in theory free in Madagascar. On average, women in the control group spent MGA 208,021 (US $48.45 according to the OANDA currency converter rate for August 16, 2022—middle of data collection) on health care during pregnancy and delivery. This amount could pose a barrier to many women who might be deterred from delivering at a health facility [8]. Given that MMHW use was associated with higher expenditure, women might actually limit their expenditure compared to their needs. As women continue to face charges for essential medication, treatment, and services beyond what is provided for free, this calls for demand-side financing solutions for maternal care.

### Related Evidence

Relative effect sizes for an increase of 11%, 32%, and 55% compared to the control group for the 3 primary outcomes (in-facility delivery, number of ANC visits, and total expenditure for health, respectively) when treatment was used as an instrument for having used the MMHW were in line with effect sizes previously reported for general demand-side and mobile health (mHealth) interventions for maternal care [28,29]. However, mHealth is broadly defined and covers a range of interventions beyond MM interventions. Evidence on MM interventions specifically is much more limited.

Several studies have assessed the association between use of MM and health outcomes. They suggest that users of MM services are more likely to use formal health care services as a response to health shocks than nonusers [30] and that their health care use may be better [31,32]. However, these studies look at MM services in general, which are mostly not focused on saving tools and are not targeted at health spending, as is the case for the MMHW. Moreover, their identification is often not causal.

mHealth technologies have become frequently used tools to address challenges related to maternal and newborn health in low- and middle-income countries [33]. Many of these technologies aimed at improving ANC access seem to have informational functions through messaging and notification alerts [34]. Few seem to have functions related to financial transactions and incentives [35] that could be directly compared to the MMHW intervention assessed in this study. A systematic review of mHealth interventions and their impact on maternal and newborn health identified 1 study evaluating an intervention with the aforementioned financial-related functions [28]. In a randomized trial in Kenya, financial incentives paid through participants’ mobile phones increased women’s probability of attending ANC, whereas they did not increase the probability of delivering at a facility [36]. This intervention was primarily a conditional cash transfer that was paid for each health visit, whereas the mobile phone was the mode of payment of the transfer. Other studies have also shown that cash transfers and vouchers can have a positive impact on the use of maternal care services [37]. In Western Kenya, a program offering a full voucher and a conditional cash transfer to expectant mothers led to an increase in institutional delivery rates by 33% [38]. Similarly, a conditional cash transfer program in Nigeria paid pregnant women to deliver at a health facility and resulted in a 41% increase in facility deliveries [39].

The MMHW was primarily a savings tool, with a conditional cash transfer in the form of vouchers and matching of deposits made by the user as an incentive to use the tool. It did not constitute a full voucher, meaning that copayment and cosaving by the user was required. This may have reduced its effectiveness compared to a full voucher [38]. Instead, this tool added an aspect to mHealth technology interventions that has not been studied previously, namely, saving. A study protocol of a randomized controlled trial in Kampala, Uganda, described the use of an MM health savings account in their intervention, but the evaluation focused on the effect of SMS text messages to encourage saving behavior and did not analyze the uptake and use of the health savings account itself [40].

The Innovative Partnership for Universal Sustainable Healthcare intervention in Kenya provided fully subsidized, digital health insurance through women’s own SIM cards to low-income women of reproductive age [41]. While saving was not a component of the intervention, the goal was to improve access to health insurance using mobile technology and increase the use of maternal health care services. All women in the treatment group received the digital health insurance on their mobile phones. This intervention did not improve maternal health care use [41].

To the best of our knowledge, this was the largest trial of a digital financing solution for maternal health. This trial was implemented in 63 CSBs and 4 reference hospitals in a central region of Madagascar. The evaluation of the intervention rigorously followed a randomized controlled trial protocol for a public health intervention. By conducting a large population-based survey instead of focusing on women visiting health care facilities, the evaluation used the highest possible bar to measure the relevant outcomes.

### Limitations

Despite these strengths, this study faced several limitations. The main concern is related to statistical power. While the original power calculation assumed uptake of 50% of women in the intervention area, actual awareness of the intervention and uptake were lower. Uptake among women who had heard of the intervention was 37.42% (485/1296) and, thus, closer to the initially anticipated uptake. We addressed the lower-than-assumed uptake by increasing the sample size of the survey during data collection. However, given an uptake of 14.51% (485/3343), we would have needed to increase the sample size 44-fold to reach a power of 0.8 compared to complete uptake (1/[compliance rate]^2^). We conducted a post hoc power analysis based on the collected data and realized uptake rate following the study by Duflo et al [42] for power calculations with grouped errors and imperfect compliance. Given the 14.51% (485/3343) registration rate in intervention areas, 31 clusters in each group, and an average of 103 respondents per cluster, we can only reject effect sizes at the population level larger than a 69–percentage point increase in delivery at a health facility, larger than an increase of 2.5 ANC visits, and larger than an increase of MGA 558,731 (US $132.80 according to the OANDA currency converter rate for August 16, 2022) in health expenditure. This study was underpowered to detect smaller yet still meaningful effects. Therefore, we cannot reject that the study did not have the observed effect. One possible approach to address low uptake and the resulting low power in future studies would be to sample eligible individuals at health care facilities, randomly assign registration among this sample, and survey only these individuals before and after the intervention. This approach does not allow for an analysis of uptake in a real-world scenario of program rollout. We particularly chose the population-based design to be as close as possible to the real-world effect of the intervention. However, we are aware that this is a composite evaluation of the intervention itself and its implementation, which could have led to the observed lower-than-anticipated uptake. Further research is needed on how the implementation could have impacted uptake, but this is out of scope for this analysis.

No baseline data collection was conducted, which would have strengthened the power of the analysis. The lack of baseline data also meant that it was not possible to assess changes over time and control for potential confounding factors. Our estimation approach solely compared outcomes between the intervention and control groups at endline and relied on the randomized treatment assignment for causal identification.

The larger effect sizes in the IV analysis suggest that the intervention might have been beneficial for MMHW users. Unfortunately, the IV analysis had even greater demands on power, and no statistical significance was achieved. Assuming that all potential users behaved similarly when registered for the tool, the local average treatment effect showed the effect under full uptake. However, this is a strong assumption that may not be valid in the study context. Women who did not register for the tool voluntarily might have behaved differently once registered than those who did. The decision to register with the MMHW may have been influenced by characteristics that are also associated with the measured outcomes or how individuals may respond to the intervention. The factors that are associated with the decision to register have been analyzed quantitatively and qualitatively elsewhere (Schäfer, L, et al., unpublished data, June 2025). Therefore, the results of the IV analysis should be interpreted with caution. Moreover, the IV analysis assumed that treatment assignment affected outcomes only through MMHW uptake. This assumption may be violated if sensitization campaigns influenced health-seeking behavior independently of registration with the MMHW. However, sensitization activities strongly focused on information about the MMHW and did not include general messages such as a call to deliver at a health facility. Therefore, it seems implausible that health-seeking behavior was influenced independently of registration.

Our analysis was an evaluation of the entire intervention package, which combined financial, patient-focused components (eg, savings incentives and free ultrasound checkups) with nonfinancial, health care provider-level components (eg, health care worker training). We are not able to clearly disentangle the effects of these different components. However, when treatment was used as an instrument for having registered with the MMHW, effect sizes were often larger than when treatment was used as an instrument for having heard of the MMHW. This indicates that the components that were dependent on registration (eg, access to free ultrasound checkups and savings incentives) were more relevant than those that were not dependent on registration (ie, health care worker training). Similarly, the larger effect sizes when treatment was used as an instrument for having used the MMHW to pay compared to having registered with the MMHW suggest that the ability to save and collect contributions using the tool may be more beneficial than the vouchers for ultrasound checkups.

Further limitations relate to intervention assignment at the individual level and sample selection into the population-based survey. The analysis treated women in the catchment area of the intervention CSBs as treated. However, these women did not necessarily attend the CSB of their *fokontany* (administrative unit) for various reasons. This CSB might not be the one closest to their residence, they might visit another CSB for other personal reasons irrespective of the CSB’s ability to offer payment through the MMHW, or they might not visit any CSB during pregnancy or for delivery. We only observed visits to the CSB after the intervention, and the decision to visit any CSB or a particular CSB might be affected by the intervention. Therefore, it is not possible to capture where women would have attended in a scenario without the intervention, and assigning treatment status through residence in the catchment area was the only option to link women with CSBs given our data.

Furthermore, the analysis was based on survey data from women who were home at the time of the visit, which took place during daytime on weekdays. These women were different from eligible women who were not present at their home, as shown previously. The latter were found to be slightly older and wealthier and have a higher probability of having access to a mobile phone. Studies of MM adoption have shown that wealthier and better educated individuals and those who own a mobile phone are more likely to adopt MM [43]. Therefore, it is possible that uptake of the MMHW might have been higher among those eligible women who were not found at their home at the time of the visit and, therefore, did not participate in the survey. While this means that we potentially underestimated intervention uptake, it is unclear how the sample selection bias impacted the estimated effects of the intervention. On the one hand, wealthier and better educated individuals who own a mobile phone could be in a better position to use the MMHW, leading to an underestimation of the effect using the available survey sample. On the other hand, slightly poorer individuals might benefit more from the MMHW as their financial barriers are more severe, resulting in an overestimation of the population-level effect. A population-based survey always faces difficulties in surveying individuals who are not present at their homes. An alternative approach would be sampling at health care facilities. However, this would lead to a different selection bias as women who do not visit health care facilities for ANC or delivery would not be included. Our study had the explicit goal of measuring the ITT effect at the population level.

A last concern relates to the quality of facility-level data based on facility records, which seemed rather imprecise, particular regarding expenses for medication and deaths during delivery. Expenses were not tracked consistently in facility records, and respondents often reported estimates, which was indicated by respondents reporting the same values for 2020 and 2021 and reporting intervals instead of a single value. Deaths of mothers or children were often not reported at all. This limits the usability of the health care provider record data. Underreported maternal and neonatal deaths could underestimate true mortality rates, thereby masking differences between facilities in the intervention and control groups. Imprecise reporting of expenses may negatively affect the precision of effect estimates on ANC and delivery drug expenses. While we followed the published protocol [26] and analyzed the facility-level data, our interpretation puts a larger weight on the data from the population-based survey.

### Conclusions

While this study did not identify a statistically significant impact, the estimated contamination-adjusted effects suggest that the MMHW has potential to improve access to maternal care for women who are receptive to such an MM-based savings tool. Estimated population-level effects were much smaller, and this study was underpowered to detect such effects due to lower-than-anticipated uptake of the intervention. It is now of great interest to investigate the reasons for low uptake and strategies to increase adoption of the MMHW or similar tools. The MMHW intervention consisted of several components that could be adjusted to increase the tool’s suitability and attractiveness to potential users. Further research could explore which information channels are most effective in informing potential users about the tool, which information sources are locally trusted by potential users to encourage uptake, and whether different incentive schemes could increase saving among users. Adding reminders to save as a function of the MMHW could potentially increase its use and, eventually, its effectiveness. On the health care providers’ side, a better understanding of their perception of the tool and their motivation for its use is required. While health care providers received monetary incentives to file claims via the MMHW platform, these incentives might have been insufficient to compensate for the perceived increased workload or adjustment to the new process. Similar interventions should include health care providers in the design of tools and use processes to ensure their acceptance of and comfort with the interventions. In conclusion, while the potential of the MMHW lies in its promise to lower barriers to accessing obstetric care, this potential is not yet fully realized.

## Appendix

Multimedia Appendix 1 Additional descriptive tables and results tables.

Multimedia Appendix 2 CONSORT-eHEALTH checklist (V 1.6.x).

## Abbreviations

ANC: antenatal care
CSB: Centres de Santé de Base
ITT: intention-to-treat
IV: instrumental variable
mHealth: mobile health
MM: mobile money
MMHW: Mobile Maternal Health Wallet

## Data Availability

The individual-level datasets analyzed during this study are available in the Göttingen Research Online repository [44]. The facility-level data analyzed during this study are not publicly available due to identification risks.
