# Supplementary material for: The Impact of a Mobile Money–Based Intervention on Maternal and Neonatal Health Outcomes in Madagascar: Cluster-Randomized Controlled Trial
Source: JMIR Public Health Surveill. 2025 Aug 15;11:e70182. doi: 10.2196/70182 (PMC12397756; doi:10.2196/70182)
Supplement: Multimedia Appendix 1 [file publichealth_v11i1e70182_app1.pdf]

The impact of a mobile money-based intervention on maternal and neonatal health outcomes in Madagascar: a cluster-randomized controlled trial

Lisa Bogler, Bítia Vieira, Harizaka Emmanuel Andriamasy, Zavaniarivo Rampanjato, Sebastian Vollmer, Till Bärnighausen, Andriamampianina Ralisimalala, Julius Valentin Emmrich*, Samuel Knauss*

Appendix

Table S 1: List of outcomes on individual level

Outcome	Description	Scale	Protocolname
Primary outcomes			
Delivery at a facility	1 if woman delivered in a health facility	binary	Facility-based delivery
Number of ANC[a] visits	Number of ANC visits	count	ANC visits
Total expenditure for health	Total expenditure for health, including medication, health checks, delivery, referrals, neonatal care (excluding extreme outlier values), in MGA	continuous	Total healthcare expenditure
Secondary outcomes			
ANC-related outcomes			
Number of ANC diagnoses received	Number of diagnoses received / danger signs detected during ANC visits	count	ANC diagnoses
Had any ultrasound checkup	1 if woman had any ultrasound check	binary	Prenatal ultrasound examination
Received iron and folic acid tablets or syrup	1 if woman received any iron and folic acid tablets or syrup during ANC visits	binary	ANC drugs received
Delivery-related outcomes			
Number of complications	Number of complications during pregnancy and delivery	count	Complications
Time to seek medical attention—immediately	1 if woman sought professional care immediately after hearing about danger sign	binary	Time to seek medical attention
Planned cesarean section	1 if delivery was a planned cesarean section	binary	Elective C-section
Emergency cesarean section	1 if delivery was an emergency cesarean section	binary	Emergency C-section
Child passed away before first birthday	1 if child passed away before first birthday	binary	Newborn mortality
Finance-related outcomes			
Amount of contributions to health savings	Amount of financial contributions for covering health care expenses from relatives or friends, in MGA	continuous	Third parties' financial contributions
Amount of health care savings	Amount saved before pregnancy or during pregnancy to cover health care expenses, in MGA	continuous	Health savings
Relative healthcare expenditure	Healthcare expenditure relative to expenditure for food/gas/electricity/water/education in past 30 days	ratio	Relative healthcare expenditure

Any indication of financial distress	1 if woman reports any sign of financial distress, including selling assets or borrowing money, reducing expenses for education or health care to cover pregnancy-related expenses, delaying or forgoing making use of pregnancy-related health care services, asking others to help cover pregnancy-related expenses	binary	Financial distress
Satisfaction-related outcomes			
Postpartum depression score	Postpartum depression score, 0 - 30	count	Postpartum depression
Satisfaction with care received at facility—fully satisfied	1 if woman was fully satisfied with care received at health facility during pregnancy/delivery	binary	Patient satisfaction
Satisfaction with how facilities are run—fully satisfied	1 if woman was fully satisfied with how health facilities are run (any experience with health facilities)	binary	Health system satisfaction
Satisfaction with health care system—fully satisfied	1 if woman was fully satisfied with how health care is run in Madagascar	binary	Health system satisfaction
Life satisfaction—fully satisfied	1 if woman was fully satisfied with life overall	binary	Life satisfaction

[a] ANC: antenatal care

Table S 2: List of outcomes on facility level

Outcome	Description	Scale	Protocolname
Number of ANC[a] visits	Number of ANC visits in 2020/2021 reported by health staff	continuous	ANC visits
Number of deliveries	Number of deliveries in 2020/2021 reported by health staff	continuous	Facility-based delivery
Maternal mortality	Number of maternal deaths divided by number of deliveries in 2020/2021 reported by health staff	rate	Maternal mortality
Newborn mortality	Number of neonatal deaths divided by number of deliveries in 2020/2021 reported by health staff	rate	Newborn mortality
Number of iron and folic acid supplements distributed	Number of iron and folic acid supplements distributed 2020/2021 as reported by health staff	continuous	ANC drugs distributed
Number of iron and folic acid supplements distributed per ANC visit	Number of iron and folic acid supplements distributed divided by number of ANC visits in 2020/2021 as reported by health staff	continuous	ANC drugs distributed
Number of anti-parasite medications distributed	Number of anti-parasite medications distributed 2020/2021 as reported by health staff	continuous	ANC drugs distributed
Expenses for ANC drugs	Expenses for ANC drugs in MGA, including iron and folic acid, anti-parasite medication	continuous	Public sector costs
Expenses for delivery drugs	Expenses for delivery drugs in MGA (GENTAMYCINE INJ/ GENTAMYCINE COLLYRE/MYSOPROSTOL/ OXYTOCINE/VITAMINE K1/ AROFOITRA/GANT/AMPICILLINE/ METRONIDAZOLE)	continuous	Public sector costs

[a] ANC: antenatal care

Table S 3: Regression results of intention-to-treat (ITT) and instrumental variable (IV) estimations for individual-level outcomes

					ITT		IV—heard of the intervention		IV—registered for the intervention		IV—used the intervention	
Outcome	Number of clusters	ICC[a]	Number of observations	Mean (SD)	Coefficient (P value)	Relative effect size	Coefficient (P value)	Relative effect size	Coefficient (P value)	Relative effect size	Coefficient (P value)	Relative effect size
Primary outcomes												
Delivery at a facility	61	0.032	6185	0.780 (0.414)	0.007 (0.7620)	0.009	0.026 (0.7563)	0.033	0.060 (0.7558)	0.077	0.086 (0.7669)	0.110
Number of ANC[b] visits	61	0.021	6186	4.325 (1.515)	0.102 (0.1518)	0.024	0.381 (0.1303)	0.088	0.901 (0.1385)	0.208	1.408 (0.1225)	0.326
Total expenditure for health (MGA)	61	0.048	4692	208021 (330946)	9309 (0.6292)	0.045	32891 (0.6299)	0.157	78346 (0.6328)	0.374	116198 (0.6428)	0.555
Secondary outcomes												
<u>ANC-related outcomes</u>												
Number of ANC diagnoses received	61	0.000	6148	0.078 (0.364)	-0.010 (0.1623)	-0.128	-0.038 (0.1410)	-0.487	-0.088 (0.1406)	-1.128	-0.131 (0.1545)	-1.679
Had any ultrasound checkup	61	0.084	6210	0.691 (0.462)	0.022 (0.5473)	0.032	0.080 (0.5486)	0.115	0.187 (0.5507)	0.269	0.280 (0.5506)	0.403
Received iron and folic acid tablets or syrup	61	0.020	6188	0.892 (0.310)	0.001 (0.9497)	0.001	-0.000 (0.9968)	0.000	-0.000 (0.9977)	0.000	0.002 (0.9908)	0.002
<u>Delivery-related outcomes</u>												
Number of complications	61	0.002	6193	0.331 (0.657)	-0.016 (0.3570)	-0.048	-0.060 (0.3478)	-0.181	-0.142 (0.3541)	-0.429	-0.213 (0.3613)	-0.644
Time to seek medical attention—immediately	57	0.051	347	0.733 (0.444)	-0.054 (0.3639)	-0.074	-0.235 (0.3741)	-0.317	-1.637 (0.4969)	-2.206	-4.291 (0.6383)	-5.783
Planned cesarean section	61	0.001	6163	0.020 (0.139)	0.002 (0.5453)	0.100	0.009 (0.5419)	0.450	0.021 (0.5481)	1.050	0.032 (0.5432)	1.600
Emergency cesarean section	61	0.012	6163	0.058 (0.234)	0.009 (0.2954)	0.155	0.034 (0.2720)	0.586	0.080 (0.2767)	1.379	0.123 (0.2746)	2.121
Child passed away before first birthday	61	0.000	6219	0.012 (0.109)	-0.001 (0.6956)	-0.083	-0.004 (0.6942)	-0.333	-0.010 (0.6937)	-0.833	-0.015 (0.6983)	-1.250
<u>Finance-related outcomes</u>												
Amount of contributions to health savings, excluding outliers (MGA)	61	0.015	1883	83683 (130155)	339 (0.9631)	0.004	2719 (0.9215)	0.033	6841 (0.9262)	0.082	10266 (0.9290)	0.123
Amount of health care savings, excluding outliers (MGA)	61	0.038	4205	216403 (274288)	14806 (0.3752)	0.068	53980 (0.3759)	0.249	129412 (0.3825)	0.596	191340 (0.3757)	0.881
Relative healthcare expenditure	47	0.018	1532	0.380 (0.207)	-0.002 (0.9053)	-0.005	-0.009 (0.8835)	-0.024	-0.027 (0.8846)	-0.071	-0.041 (0.8919)	-0.108

Any indication of financial distress	61	0.008	6177	0.615<br>(0.487)	-0.019<br>(0.2815)	-0.031	-0.072<br>(0.2541)	-0.117	-0.171<br>(0.2422)	-0.279	-0.258<br>(0.2431)	-0.420
<u>Satisfaction-related outcomes</u>											
Postpartum depression score	61	0.007	6070	6.331<br>(5.713)	0.017<br>(0.9283)	0.003	0.102<br>(0.8838)	0.016	0.225<br>(0.8909)	0.036	0.308<br>(0.9010)	0.049
Satisfaction with care received at facility—fully satisfied	61	0.013	6001	0.774<br>(0.418)	0.013<br>(0.4258)	0.017	0.046<br>(0.4250)	0.059	0.108<br>(0.4091)	0.139	0.162<br>(0.4002)	0.209
Satisfaction with how facilities are run—fully satisfied	61	0.016	5846	0.467<br>(0.499)	0.021<br>(0.3330)	0.045	0.078<br>(0.3203)	0.167	0.184<br>(0.3147)	0.393	0.280<br>(0.3109)	0.598
Satisfaction with health care system—fully satisfied	61	0.017	5231	0.268<br>(0.443)	0.010<br>(0.6266)	0.037	0.037<br>(0.6171)	0.139	0.086<br>(0.6135)	0.322	0.130<br>(0.6018)	0.487
Life satisfaction—fully satisfied	61	0.001	6212	0.234<br>(0.423)	-0.000<br>(0.9771)	0.000	-0.001<br>(0.9725)	-0.004	-0.003<br>(0.9703)	-0.013	0.001<br>(0.9958)	0.004

Note: This table shows the regression results of the ITT and IV estimations for primary and secondary outcomes. It reports the number of clusters and observations used for each outcome, the intracluster correlation coefficient, the mean value and standard deviation of each outcome in the control group, the coefficient of being in the catchment area of a treated *Centre de Santé de Base* (CSB; public sector primary care health facility) with the corresponding *P* value, and the relative effect size (calculated as the coefficient divided by the mean) for both ITT and IV models. In all models, indicator variables for strata were included as controls, and SEs were clustered at the CSB level.

[a]ICC: intraclass correlation coefficient.

[b]ANC: antenatal care.

Table S 4: Regression results of intention-to-treat (ITT) and instrumental variable (IV) estimations for individual-level outcomes – robustness to sample

Outcome	Number of clusters	Number of observations	Mean (SD)	ITT		IV—heard of the intervention		IV—registered for the intervention		IV—used the intervention	
				Coefficient (P value)	Relative effect size	Coefficient (P value)	Relative effect size	Coefficient (P value)	Relative effect size	Coefficient (P value)	Relative effect size
Primary outcomes											
Delivery at a facility	58	5862	0.780 (0.414)	0.021 (0.2998)	0.027	0.076 (0.2827)	0.097	0.177 (0.2850)	0.227	0.259 (0.2876)	0.332
Number of ANC[a] visits	58	5865	4.325 (1.515)	0.121 (0.0839)	0.028	0.439 (0.0654)	0.102	1.025 (0.0758)	0.237	1.583 (0.0660)	0.366
Total expenditure for health (MGA)	58	4433	208021 (330946)	18653 (0.3149)	0.090	64776 (0.3110)	0.309	150443 (0.3257)	0.718	223648 (0.3390)	1.067
Secondary outcomes											
<u>ANC-related outcomes</u>											
Number of ANC diagnoses received	58	5828	0.078 (0.364)	-0.009 (0.2124)	-0.115	-0.033 (0.1899)	-0.423	-0.076 (0.1896)	-0.974	-0.112 (0.2043)	-1.436
Had any ultrasound checkup	58	5888	0.691 (0.462)	0.028 (0.4443)	0.041	0.099 (0.4442)	0.143	0.230 (0.4489)	0.331	0.342 (0.4481)	0.493
Received iron and folic acid tablets or syrup	58	5866	0.892 (0.310)	0.001 (0.9187)	0.001	0.002 (0.9688)	0.002	0.004 (0.9683)	0.004	0.008 (0.9570)	0.009
<u>Delivery-related outcomes</u>											
Number of complications	58	5870	0.331 (0.657)	-0.019 (0.2771)	-0.057	-0.070 (0.2679)	-0.211	-0.163 (0.2781)	-0.492	-0.241 (0.2863)	-0.728
Time to seek medical attention—immediately	54	326	0.733 (0.444)	-0.075 (0.2062)	-0.102	-0.328 (0.2340)	-0.442	-1.830 (0.3884)	-2.466	-4.094 (0.5194)	-5.518
Planned cesarean section	58	5840	0.020 (0.139)	0.003 (0.4980)	0.150	0.010 (0.4938)	0.500	0.024 (0.5017)	1.200	0.036 (0.4976)	1.800
Emergency cesarean section	58	5840	0.058 (0.234)	0.011 (0.1826)	0.190	0.041 (0.1716)	0.707	0.095 (0.1803)	1.638	0.143 (0.1823)	2.466
Child passed away before first birthday	58	5897	0.012 (0.109)	-0.000 (0.9708)	0.000	-0.000 (0.9701)	0.000	-0.001 (0.9700)	-0.083	-0.001 (0.9764)	-0.083
<u>Finance-related outcomes</u>											
Amount of contributions to health savings, excluding outliers (MGA)	58	1789	83683 (130155)	248 (0.9728)	0.003	2150 (0.9348)	0.026	5427 (0.9390)	0.065	7927 (0.9424)	0.095
Amount of health care savings, excluding outliers (MGA)	58	3982	216403 (274288)	20850 (0.1994)	0.096	73438 (0.2070)	0.338	173604 (0.2212)	0.799	253980 (0.2213)	1.170
Relative healthcare expenditure	46	1420	0.380 (0.207)	0.004 (0.7766)	0.011	0.018 (0.7839)	0.047	0.051 (0.7802)	0.134	0.081 (0.7721)	0.213

Any indication of financial distress	58	5856	0.615	-0.016	-0.026	-0.059	-0.096	-0.139	-0.226	-0.208	-0.339
			(0.487)	(0.3861)		(0.3554)		(0.3416)		(0.3418)	
<u>Satisfaction-related outcomes</u>											
Postpartum depression score	58	5757	6.331	0.072	0.011	0.296	0.047	0.668	0.106	0.965	0.153
			(5.713)	(0.7051)		(0.6657)		(0.6773)		(0.6884)	
Satisfaction with care received at	58	5693	0.774	0.010	0.013	0.034	0.044	0.080	0.103	0.119	0.154
facility—fully satisfied			(0.418)	(0.5615)		(0.5598)		(0.5422)		(0.5368)	
Satisfaction with how facilities are run—	58	5553	0.467	0.016	0.034	0.058	0.124	0.135	0.288	0.202	0.432
fully satisfied			(0.499)	(0.4674)		(0.4544)		(0.4487)		(0.4453)	
Satisfaction with health care system—	58	4973	0.268	0.009	0.034	0.034	0.127	0.077	0.288	0.118	0.442
fully satisfied			(0.443)	(0.6531)		(0.6442)		(0.6400)		(0.6281)	
Life satisfaction—fully satisfied	58	5890	0.234	0.000	0.000	0.002	0.009	0.004	0.017	0.012	0.051
			(0.423)	(0.9632)		(0.9630)		(0.9641)		(0.9283)	

Note: This table shows the regression results of the ITT and IV estimations for primary and secondary outcomes. It reports the number of clusters and observations used for each outcome, the mean value and standard deviation of each outcome in the control group, the coefficient of being in the catchment area of a treated *Centre de Santé de Base* (CSB; public sector primary care health facility) with the corresponding P value in parentheses and the relative effect size (calculated as the coefficient divided by the mean) for both ITT and IV models. In all models, indicator variables for strata were included as controls, and standard errors were clustered at the CSB level. The sample excludes CSBs that were assigned to intervention group but could not implement it due to technical issues.

[a] ANC: antenatal care

Table S 5: Regression results of intention-to-treat (ITT) and instrumental variable (IV) estimations for individual-level outcomes – robustness to controls

Outcome	Number of clusters	Number of observations	Mean (SD)	ITT		IV—heard of the intervention		IV—registered for the intervention		IV—used the intervention	
				Coefficient (P value)	Relative effect size	Coefficient (P value)	Relative effect size	Coefficient (P value)	Relative effect size	Coefficient (P value)	Relative effect size
Primary outcomes											
Delivery in facility	61	6163	0.781 (0.413)	-0.005 (0.8200)	-0.006	-0.018 (0.8147)	-0.023	-0.042 (0.8156)	-0.054	-0.066 (0.8044)	-0.085
Number of ANC[a] visits	61	6164	4.328 (1.515)	0.044 (0.4366)	0.010	0.161 (0.4259)	0.037	0.374 (0.4263)	0.086	0.600 (0.3884)	0.139
Total expenditure for health (MGA)	61	4675	209844 (332703)	-6271 (0.6421)	-0.030	-23226 (0.6281)	-0.111	-54607 (0.6287)	-0.260	-82641 (0.6253)	-0.393
Secondary outcomes											
<u>ANC-related outcomes</u>											
Number of ANC diagnoses received	61	6124	0.078 (0.365)	-0.015 (0.0374)	-0.192	-0.057 (0.0238)	-0.731	-0.131 (0.0279)	-1.679	-0.192 (0.0343)	-2.462
Had any ultrasound check	61	6188	0.694 (0.461)	-0.006 (0.8201)	-0.009	-0.025 (0.7942)	-0.036	-0.058 (0.7948)	-0.084	-0.086 (0.7942)	-0.124
Received iron and folic acid tablets or syrup	61	6166	0.893 (0.309)	-0.005 (0.6878)	-0.006	-0.020 (0.6435)	-0.022	-0.047 (0.6476)	-0.053	-0.068 (0.6613)	-0.076
<u>Delivery-related outcomes</u>											
Number of complications	61	6169	0.330 (0.658)	-0.019 (0.2834)	-0.058	-0.071 (0.2732)	-0.215	-0.165 (0.2854)	-0.498	-0.243 (0.2910)	-0.734
Time to seek medical attention: immediately	57	347	0.742 (0.439)	-0.054 (0.3836)	-0.073	-0.227 (0.3801)	-0.306	-1.760 (0.4932)	-2.372	-3.442 (0.5748)	-4.639
Planned C-section	61	6141	0.019 (0.138)	-0.000 (0.9569)	0.000	-0.001 (0.9507)	-0.053	-0.002 (0.9502)	-0.105	-0.002 (0.9586)	-0.105
Emergency C-section	61	6141	0.059 (0.235)	0.002 (0.7300)	0.034	0.010 (0.6880)	0.172	0.024 (0.6886)	0.414	0.038 (0.6774)	0.655
Child passed away before 1st birthday	61	6197	0.012 (0.108)	-0.001 (0.7104)	-0.083	-0.004 (0.7089)	-0.333	-0.010 (0.7081)	-0.833	-0.014 (0.7124)	-1.167
<u>Finance-related outcomes</u>											
Amount of contributions to health savings, excluding outlier (MGA)	61	1873	84128 (130879)	-1760 (0.7940)	-0.021	-5403 (0.8345)	-0.064	-15301 (0.8263)	-0.182	-23852 (0.8246)	-0.284
Amount of health care savings, excluding outliers (MGA)	61	4191	217299 (275250)	-1728 (0.8795)	-0.008	-6332 (0.8774)	-0.029	-14604 (0.8795)	-0.067	-15840 (0.9079)	-0.073
Relative healthcare expenditure	47	1531	0.380 (0.207)	0.000 (0.9710)	0.000	0.001 (0.9862)	0.003	0.003 (0.9861)	0.008	0.007 (0.9808)	0.018

Any indication of financial distress	61	6157	0.614	-0.013	-0.021	-0.051	-0.083	-0.122	-0.199	-0.181	-0.295
			(0.487)	(0.3852)		(0.3500)		(0.3327)		(0.3324)	
<u>Satisfaction-related outcomes</u>											
Postpartum depression score	61	6049	6.316	-0.070	-0.011	-0.225	-0.036	-0.537	-0.085	-0.813	-0.129
			(5.694)	(0.6773)		(0.7120)		(0.7038)		(0.6971)	
Satisfaction with care received at facility—fully satisfied	61	5980	0.776	0.015	0.019	0.052	0.067	0.122	0.157	0.178	0.229
			(0.417)	(0.3582)		(0.3482)		(0.3319)		(0.3246)	
Satisfaction with how facilities are run—fully satisfied	61	5826	0.468	0.031	0.066	0.114	0.244	0.266	0.568	0.398	0.850
			(0.499)	(0.1231)		(0.1111)		(0.1096)		(0.1087)	
Satisfaction with health care system—fully satisfied	61	5216	0.267	0.019	0.071	0.071	0.266	0.162	0.607	0.240	0.899
			(0.443)	(0.3210)		(0.3166)		(0.3177)		(0.3070)	
Life satisfaction—fully satisfied	61	6190	0.235	-0.001	-0.004	-0.005	-0.021	-0.012	-0.051	-0.012	-0.051
			(0.424)	(0.9084)		(0.9019)		(0.8989)		(0.9308)	

Note: This table shows the regression results of the ITT and IV estimations for primary and secondary outcomes, controlling for employment type, ownership of phone, television, and bicycle. It reports the number of clusters and observations used for each outcome, the mean value and standard deviation of each outcome in the control group, the coefficient of being in the catchment area of a treated *Centre de Santé de Base* (CSB; public sector primary care health facility) with the corresponding P value in parentheses and the relative effect size (calculated as the coefficient divided by the mean) for both ITT and IV models. In all models, indicator variables for strata were included as controls, and standard errors were clustered at the CSB level.

[a] ANC: antenatal care

Table S 6: Regression results of intention-to-treat (ITT) estimations for facility-level outcomes – robustness to sample

Outcome	ITT			
	Number of observations	Mean (SD)	Coefficient (P value)	Relative effect size
<u>Outcomes based on 2020 records</u>				
Number of ANC[a] visits	54	1582 (1102)	132 (0.4612)	0.087
Number of deliveries	53	241 (265)	56 (0.3131)	0.236
Maternal mortality	37	0.004 (0)	0.002 (0.3656)	0.667
Newborn mortality	36	0.000 (0)	-0.001 (0.3683)	—[b]
Number of iron and folic acid supplements distributed	52	39186 (22016)	2350 (0.6936)	0.059
Number of iron and folic acid supplements distributed per ANC visit	52	28 (2)	-10 (0.4417)	-0.303
Number of anti-parasite medications distributed	53	323 (233)	-0 (0.9949)	0.000
Expenses for ANC drugs (MGA)	54	1490 (219)	83 (0.1172)	0.057
Expenses for delivery drugs (MGA)	38	9167 (5706)	2732 (0.0453)	0.343
<u>Outcomes based on 2021 records</u>				
Number of ANC visits	53	1627 (1175)	254 (0.1453)	0.176
Number of deliveries	47	238 (258)	59 (0.2246)	0.271
Maternal mortality	34	0.004 (0)	-0.004 (0.4934)	-0.667
Newborn mortality	35	0.000 (0)	0.000 (.)	—
Number of iron and folic acid supplements distributed	49	40427 (23978)	-616 (0.9030)	-0.015
Number of iron and folic acid supplements distributed per ANC visit	49	28 (2)	-8 (0.3623)	-0.250
Number of anti-parasite medications distributed	49	341 (255)	3 (0.9652)	0.009
Expenses for ANC drugs (MGA)	48	1477 (212)	55 (0.3351)	0.038
Expenses for delivery drugs (MGA)	33	8541 (5207)	1270 (0.2963)	0.164

Note: This table shows the regression results of the ITT estimations for the secondary outcomes on facility level. It reports the number of observations used for each outcome, the mean value of each outcome in the control group, the coefficient of being treated with the corresponding P value in parentheses and the relative effect size (calculated as the coefficient divided by the mean). Indicator variables for strata were included as controls. The sample excludes *Centres de Santé de Base* (CSBs; public sector primary care health facilities) and reference hospitals that were assigned to the intervention group non-randomly and it excludes CSBs that were assigned to the intervention group randomly but could not implement it due to technical issues.

[a] ANC: antenatal care

[b] Cell is empty because relative effect size could not be calculated given the mean of 0.000.
