# Supplementary material for: The Impact of a Mobile Money–Based Intervention on Maternal and Neonatal Health Outcomes in Madagascar: Cluster-Randomized Controlled Trial
Source: JMIR Public Health Surveill. 2025 Aug 15;11:e70182. doi: 10.2196/70182 (PMC12397756; doi:10.2196/70182)
Supplement: Multimedia Appendix 2 [file publichealth_v11i1e70182_app2.pdf]

CONSORT 2010 checklist of information to include when reporting a randomised trial*

Section/Topic	Item No	Standard checklist item	Extension for cluster designs	Reported on page No
**Title and abstract**				
	1a	Identification as a randomised trial in the title	Identification as a cluster randomized trial in the title	1
	1b	Structured summary of trial design, methods, results, and conclusions (for specific guidance see CONSORT for abstracts)		1
**Introduction**				
Background and objectives	2a	Scientific background and explanation of rationale	Rationale for using a cluster design	2 (intervention on facility level)
	2b	Specific objectives or hypotheses	Whether objectives pertain to the cluster level, the individual participant level, or both	3 (impact on maternal and neonatal health outcomes)
**Methods**				
Trial design	3a	Description of trial design (such as parallel, factorial) including allocation ratio	Definition of cluster and description of how the design features apply to the clusters	3
	3b	Important changes to methods after trial commencement (such as eligibility criteria), with reasons		3 (section Randomization and Masking)
Participants	4a	Eligibility criteria for participants	Eligibility criteria for clusters	3
	4b	Settings and locations where the data were collected		3
Interventions	5	The interventions for each group with sufficient details to allow replication, including how and when they were actually administered	Whether interventions pertain to the cluster level, the individual participant level, or both	4 (section Procedures)
Outcomes	6a	Completely defined pre-specified primary and secondary outcome measures, including how and when they were assessed	Whether outcome measures pertain to the cluster level, the individual participant level, or both	4 (section Outcomes)
	6b	Any changes to trial outcomes after the trial commenced, with reasons		NA

Sample size	7a	How sample size was determined	Method of calculation, number of cluster(s) (and whether equal or unequal cluster sizes are assumed), cluster size, a coefficient of intracluster correlation (ICC or k) and an indication of its uncertainty	5 (section Power Calculations)
	7b	When applicable, explanation of any interim analyses and stopping guidelines		NA
Randomisation:				
Sequence generation	8a	Method used to generate the random allocation sequence		3 (section Randomization and masking)
	8b	Type of randomisation; details of any restriction (such as blocking and block size)	Details of stratification or matching if used	3 (section Randomization and masking)
Allocation concealme nt mechanism	9	Mechanism used to implement the random allocation sequence (such as sequentially numbered containers), describing any steps taken to conceal the sequence until interventions were assigned	Specification that allocation was based on clusters rather than individuals and whether allocation concealment (if any) was at the cluster level, the individual participant level, or both	3 (section Randomization and masking)
Implementation	10	Who generated the random allocation sequence, who enrolled participants, and who assigned participants to interventions	Replaced by 10a, 10b, and 10c	-
	10a		Who generated the random allocation sequence, who enrolled clusters, and who assigned clusters to interventions	3 (section Randomization and masking)
	10b		Mechanism by which individual participants were included in clusters for the purposes of the trial (such as complete enumeration, random sampling)	3 (section Participants)
	10c		From whom consent was sought (representatives of the cluster, or individual cluster members, or both) and whether consent was sought before or after randomization	-
Blinding	11a	If done, who was blinded after assignment to interventions (for example, participants, care		4 (section Randomization and

		providers, those assessing outcomes) and how		Masking)
	11b	If relevant, description of the similarity of interventions		NA
Statistical methods	12a	Statistical methods used to compare groups for primary and secondary outcomes	How clustering was taken into account	4 (section Statistical Analysis)
	12b	Methods for additional analyses, such as subgroup analyses and adjusted analyses		4 (section Statistical Analysis)
**Results**				
Participant flow (a diagram is strongly recommended)	13a	For each group, the numbers of participants who were randomly assigned, received intended treatment, and were analysed for the primary outcome	For each group, the number of clusters that were randomly assigned, received intended treatment, and were analysed for the primary outcome	8 (section Uptake of the MMHW Intervention)
	13b	For each group, losses and exclusions after randomisation, together with reasons	For each group, losses and exclusions for both clusters and individual cluster members	8 and 3
Recruitment	14a	Dates defining the periods of recruitment and follow-up		3 (section Participants)
	14b	Why the trial ended or was stopped		NA
Baseline data	15	A table showing baseline demographic and clinical characteristics for each group	Baseline characteristics for the individual and cluster levels as applicable for each group	Table 1, Table 2
Numbers analysed	16	For each group, number of participants (denominator) included in each analysis and whether the analysis was by original assigned groups	For each group, number of clusters included in each analysis	Table 3, Table S3
Outcomes and estimation	17a	For each primary and secondary outcome, results for each group, and the estimated effect size and its precision (such as 95% confidence interval)	Results at the individual or cluster level as applicable and a coefficient of intracluster correlation (ICC) for each primary outcome	Table 3 and Table S3 (individual level), Table 4 (cluster level)
	17b	For binary outcomes, presentation of both absolute and relative effect sizes is recommended		Tables 3, S3 and 4 include both
Ancillary analyses	18	Results of any other analyses performed, including subgroup analyses and adjusted analyses, distinguishing pre-specified from		Tables S4, S5, S6

		exploratory		
Harms	19	All important harms or unintended effects in each group (for specific guidance see CONSORT for harms)		-
**Discussion**				
Limitations	20	Trial limitations, addressing sources of potential bias, imprecision, and, if relevant, multiplicity of analyses		15-16 (section Limitations)
Generalisability	21	Generalisability (external validity, applicability) of the trial findings	Generalisability to clusters and/or participants (as relevant)	15-16
Interpretation	22	Interpretation consistent with results, balancing benefits and harms, and considering other relevant evidence		14
**Other information**				
Registration	23	Registration number and name of trial registry		1 (Abstract)
Protocol	24	Where the full trial protocol can be accessed, if available		4 (reference to study protocol 26)
Funding	25	Sources of funding and other support (such as supply of drugs), role of funders		17 (section Acknowledgments)

Citation: Schulz KF, Altman DG, Moher D, for the CONSORT Group. CONSORT 2010 Statement: updated guidelines for reporting parallel group randomised trials. BMC Medicine. 2010;8:18.
© 2010 Schulz et al. This is an Open Access article distributed under the terms of the Creative Commons Attribution License (http://creativecommons.org/licenses/by/2.0), which permits unrestricted use, distribution, and reproduction in any medium, provided the original work is properly cited.

*We strongly recommend reading this statement in conjunction with the CONSORT 2010 Explanation and Elaboration for important clarifications on all the items. If relevant, we also recommend reading CONSORT extensions for cluster randomised trials, non-inferiority and equivalence trials, non-pharmacological treatments, herbal interventions, and pragmatic trials. Additional extensions are forthcoming: for those and for up-to-date references relevant to this checklist, see www.consort-statement.org.
